# Ultrafast Transient Absorption Spectra and Kinetics of Rod and Cone Visual Pigments

**DOI:** 10.3390/molecules28155829

**Published:** 2023-08-02

**Authors:** Arjun Krishnamoorthi, Keyvan Khosh Abady, Dinesh Dhankhar, Peter M. Rentzepis

**Affiliations:** 1 Department of Electrical and Computer Engineering, Texas A&M University, College Station, TX 77843, USA; 2 Thermo Fisher Scientific, Hillsboro, OR 97124, USA

**Keywords:** photoreceptors, phototransduction, photo-intermediates, opsins, transient absorption

## Abstract

Rods and cones are the photoreceptor cells containing the visual pigment proteins that initiate visual phototransduction following the absorption of a photon. Photon absorption induces the photochemical transformation of a visual pigment, which results in the sequential formation of distinct photo-intermediate species on the femtosecond to millisecond timescales, whereupon a visual electrical signal is generated and transmitted to the brain. Time-resolved spectroscopic studies of the rod and cone photo-intermediaries enable the detailed understanding of initial events in vision, namely the key differences that underlie the functionally distinct scotopic (rod) and photopic (cone) visual systems. In this paper, we review our recent ultrafast (picoseconds to milliseconds) transient absorption studies of rod and cone visual pigments with a detailed comparison of the transient molecular spectra and kinetics of their respective photo-intermediaries. Key results include the characterization of the porphyropsin (carp fish rhodopsin) and human green-cone opsin photobleaching sequences, which show significant spectral and kinetic differences when compared against that of bovine rhodopsin. These results altogether reveal a rather strong interplay between the visual pigment structure and its corresponding photobleaching sequence, and relevant outstanding questions that will be further investigated through a forthcoming study of the human blue-cone visual pigment are discussed.

## 1. Introduction

Vision in humans and other vertebrates is mediated by the rod and cone photoreceptor cells, which comprise the retina of the visual system and contain the visual pigment proteins necessary for sensing light [1,2,3]. In the human retina, there are about 20 times more rods than cones [1,4], and several important functional differences exist between them. Notably, the rods are capable of detecting a single photon [2,5,6], thus displaying remarkable photosensitivity, whereas the cones are significantly less photosensitive (generally requiring hundreds of photons for activation [4,7]) but can respond to successive light stimuli faster, along with having more effective brightness adaptation, than the rods [2,7,8,9]. Furthermore, cone cells provide higher spatial acuity (resolution) due to their less convergent neural circuitry [4] and are thus more concentrated near the fovea of the retina, whereas the rod cells are mostly located at the periphery [2]. Consequently, rod cells are responsible for scotopic (dim light or night) vision due to their superior photosensitivity, whereas cone cells provide photopic (bright light or daylight) vision characterized by higher spatial, temporal, and spectral resolution [2,3,4,7,8,9,10,11,12,13]. In particular, the visual systems of humans and related primates contain a single type of rod cell, along with three distinct types of cone cells that altogether enable trichromatic color vision [2,4,8,14].

**Figure 1:**
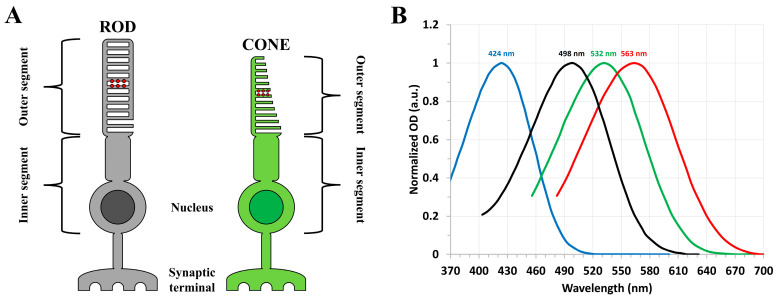
(**A**) Morphology of rod (left) and cone (right) photoreceptor cells, where the outer segment portions contain the distinct visual pigment proteins represented by the red circles. (**B**) Normalized steady-state absorption spectra of bovine rhodopsin (black) and the human cone (red, green, and blue) visual pigments with the approximate absorption band maxima indicated. Spectra are plotted based on the data in [1,8].

Morphologically, both the rods and cones consist of outer segments [1,2,13], as displayed in Figure 1A, that contain the distinct visual pigment proteins required for the absorption of light and subsequent visual sensation. The rod and cone visual pigments consist primarily of an 11-*cis* retinal chromophore (prosthetic group) covalently bound to an opsin apoprotein by means of a protonated Schiff base linkage [1,2,10,15,16,17]. Differences in the primary structures of the rod and cone opsin apoproteins result in the unique spectral response of each photoreceptor cell type [2,8,18], as shown in Figure 1B. For humans, the single rod visual pigment (rhodopsin) displays maximum absorption at ~500 nm [1], while the three types of cone visual pigments (cone opsins) most efficiently absorb in the red (~563 nm), green (~532 nm), and blue (~424 nm) spectral regions, depending on the cone cell type [8]. Substitutions in the configuration of the retinal chromophore can also significantly modify the spectral response of the resultant visual pigment [3,19,20,21].

Rod and cone visual pigments are members of the family of integral membrane proteins known as G protein-coupled receptors [16], which initiate signaling cascades in response to an external stimulus. The visual process (phototransduction) begins in a photoreceptor cell through the absorption of a photon by its corresponding visual pigment protein, which results in the ultrafast, structural isomerization of the retinal chromophore from an 11-*cis* to all-*trans* configuration [1,2,11,15]. This rapid distortion of the chromophore molecular structure induces an ordered sequence of conformational changes in the opsin apoprotein, thereby resulting in the formation and decay of several distinct photo-intermediate species on timescales ranging from femtoseconds to milliseconds [1,15,16,22,23]. Of particular importance is the formation of the final metastable, G protein-activating, state known as metarhodopsin II [1,3,11,16,22,24], which binds to and activates the associated G protein (guanine nucleotide-binding protein) transducin prior to the eventual dissociation of the all-*trans* retinal chromophore from the opsin apoprotein, thus triggering a series of enzymatic reactions that ultimately produce the visual electrical signal transmitted to the brain [2,9,11,12,13,16,25]. While the general mechanism of phototransduction is similar between the rods and cones [9,12], differences in the G protein activation efficiency, along with the rates of inactivation and subsequent regeneration [26], of the rod and cone visual pigments result in the higher, single-photon sensitivity of rod cells, along with the superior temporal resolution of cone cells owing to their shorter flash (electrical) response [9,12,24,27]. Such differences may be partly ascribed to the differing morphologies of the rod and cone outer segments [4], as displayed in Figure 1A.

The steady-state absorption spectra of the human rod and cone visual pigments were recorded, first in solution and then in retinal cells, through spectrophotometric techniques as early as the 1930s [28,29,30,31,32,33,34], and low temperature studies [35,36,37] revealed the distinct photo-intermediate species formed following photon absorption. The development of the flash photolysis method [38,39], along with the subsequent invention of the laser and pump–probe spectroscopic technique [40,41] in the 1960s, enabled the recording of the transient molecular spectra and kinetics of photo-excited rhodopsin with unprecedented temporal resolution, thus providing substantial knowledge regarding its photobleaching sequence on the femtosecond to millisecond timescales [22,23,42,43,44,45,46,47,48,49,50]. Notably, low-temperature picosecond spectroscopic studies [51,52] revealed the ultrafast formation of the first metastable photo-intermediate, namely bathorhodopsin (Batho), and suggested that quantum (proton) tunneling of the Schiff base H^+^ is an operative photochemical mechanism at low temperatures. Subsequent studies [23,24,27,53,54,55,56] have detected and characterized the lumirhodopsin (Lumi) and metarhodopsin I/II (Meta-I/II) photo-intermediates formed on the nanosecond to millisecond timescales, thus providing a complete spectral and kinetic description of the rhodopsin photobleaching sequence prior to G protein activation. Lately, femtosecond optical pump pulses have been employed in conjunction with femtosecond X-ray probe pulses (generated from an X-ray free-electron laser) to study the ultrafast *cis*-to-*trans* isomerization of the retinal chromophore, along with the subsequent conformational changes in the opsin apoprotein required for bathorhodopsin formation, in crystallized rhodopsin with atomic spatial resolution [57]. In contrast to rod cells, cone cells have been studied in considerably less detail, owing to their limited availability and stability upon purification [8]. While electrophysiological and optical measurements (typically on the millisecond to second, or even minute, timescales [24,58]) have provided important biochemical information regarding differences in the photosensitivity and recovery of the rod and cone visual pigments following G protein activation [4,9,12,13,24,27], equivalent pump–probe optical studies of the cone visual pigments with sub-millisecond temporal resolution are greatly limited [59,60,61]; hence, our understanding of the photochemical transformation of cone visual pigments is rather low. Consequently, detailed knowledge regarding the transient molecular spectra and kinetics of cone photo-intermediates, namely how they compare between the various cone cell types and against those of rhodopsin, is fundamentally lacking. In addition to providing basic understanding about cone-mediated phototransduction, such information would likely help explain important functional differences between the rods and cones.

In this paper, we summarize our recent ultrafast transient absorption studies [62,63] of the rod and cone visual pigments at room temperature. We first describe experimental results demonstrating spectral and kinetic differences in the photobleaching sequences of the two primary rod visual pigments found in vertebrates, namely rhodopsin and porphyropsin, where the latter is commonly found in aquatic life such as fishes and frogs [20]. While rhodopsin has been extensively studied over the past several decades, cone opsins are significantly less characterized, and this has resulted in comparatively limited knowledge about cone-mediated vision. Recent advances in the synthesis, purification, and stabilization of human cone visual pigments obtained through recombinant techniques [64] enabled us to perform similar pump–probe measurements on the human green-cone visual pigment for the first time, which is particularly notable given that very few ultrafast studies have been conducted on cone visual pigments. The time-resolved spectroscopic systems employed are described, and we briefly outline our ongoing work in investigating the human blue-cone visual pigment, which will provide critical insight regarding differences in the phototransduction mechanisms of the various cone cell types. These ultrafast experimental results provide a means for further understanding and comparing rod- and cone-mediated vision at the fundamental molecular level, namely with respect to the transient absorption spectra and kinetics of the photo-intermediate states formed following photon absorption, which are among the initial events in the visual process.

## 2. Results

### 2.1. Bovine Rhodopsin

**Figure 2:**
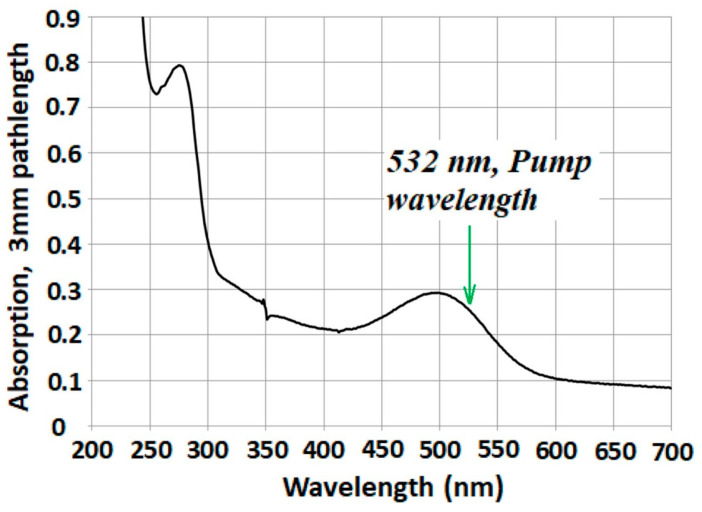
Steady-state absorption spectrum of bovine rhodopsin solubilized in a detergent-buffer medium. The excitation wavelength employed for pumping is indicated by the arrow. Figure is reproduced from [62].

We first performed time-resolved absorption experiments on bovine rhodopsin, which was extracted from rod outer segments (ROS) and solubilized in a detergent-buffer medium. Figure 2 displays the steady-state absorption spectrum of the solubilized bovine rhodopsin, which shows a rather broad absorption band centered at ~500 nm.

**Figure 3:**
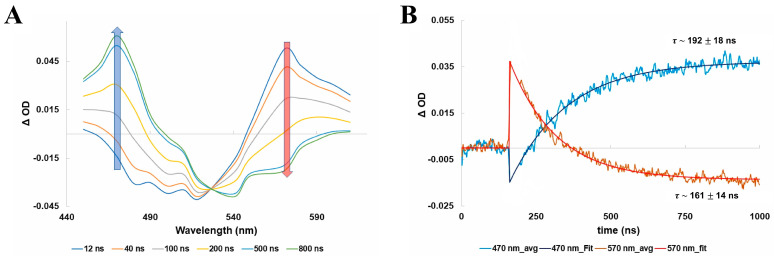
(**A**) Nanosecond transient absorption spectra of bovine rhodopsin following optical excitation with a nanosecond laser pulse at 532 nm. (**B**) Kinetic traces and exponential fits (assuming first−order kinetics) at 570 nm and 470 nm, corresponding to the decay of bathorhodopsin and formation of lumirhodopsin, respectively. Note that the first ~35 ns of the recorded kinetic traces, following excitation, are excluded from the exponential fittings due to the photodetector response. Figures are reproduced with some modifications from [63].

Following the optical excitation of bovine rhodopsin with a 532 nm nanosecond laser pulse at room temperature, the first metastable photo-intermediate bathorhodopsin is formed within ~1 ps after the initial *cis*-to-*trans* isomerization of the retinal chromophore within ~200 fs [22,47,48,49,50,57], which is faster than our experimental time resolution. We can, however, resolve the decay of bathorhodopsin and subsequent formation of lumirhodopsin, which occurs on the nanosecond timescale. This is shown in Figure 3, which displays the nanosecond transient absorption spectra of bovine rhodopsin, along with the corresponding kinetic traces for monitoring the decay of bathorhodopsin and formation of lumirhodopsin.

**Figure 4:**
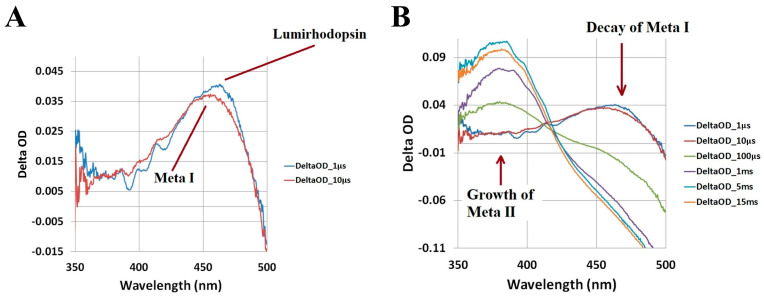
Microsecond to millisecond transient absorption spectra of bovine rhodopsin following optical excitation with a nanosecond laser pulse at 532 nm. The transient absorption spectra illustrate (**A**) the conversion of lumirhodopsin into metarhodopsin I within ~10 µs and (**B**) subsequent decay of metarhodopsin I, which results in the formation of metarhodopsin II. Figures are reproduced with some modifications from [62].

The nanosecond transient absorption spectra shown in Figure 3A display the characteristic ground-state bleach signal of bovine rhodopsin centered at ~500 nm, along with the formation band centered at ~570 nm that is attributed to the first metastable intermediate bathorhodopsin. The bathorhodopsin absorption band subsequently decays in time and feeds the growth of the lumirhodopsin absorption band centered at ~470 nm. Kinetic analysis of the transient absorption spectra, as provided in Figure 3B, demonstrates that both the decay of bathorhodopsin and formation of lumirhodopsin follow first-order kinetics with comparable time constants (within the experimental error), which further supports the direct interconversion of bathorhodopsin into lumirhodopsin (fully formed within ~1 µs following excitation). Probing photo-excited bovine rhodopsin at microsecond and millisecond delay times allowed us to further observe the conversion of lumirhodopsin into metarhodopsin I, along with the eventual formation of the final metastable photo-intermediate metarhodopsin II, as displayed in Figure 4.

As shown in Figure 4A, lumirhodopsin is fully converted into metarhodopsin I within ~10 µs. We identify metarhodopsin I based on its characteristic blue-shifted (by ~10 nm) absorption band relative to lumirhodopsin [1,15,23,56]. Consequently, the ~460 nm absorption band of metarhodopsin I decays and feeds the formation of another blue-shifted, ~380 nm, absorption band corresponding to the G protein-activating metarhodopsin II photo-intermediate, as displayed in Figure 4B. The conversion of metarhodopsin I into metarhodopsin II is associated with the deprotonation of the Schiff base linkage between the all-*trans* retinal chromophore and opsin apoprotein [1,15], which results in the significantly blue-shifted absorption band maximum. Based on the time-resolved spectral data shown in Figure 4, we determined that metarhodopsin II is formed within ~1 ms, which agrees with previous studies [1,15,17,22,23,65]. As previously stated, metarhodopsin II represents the final metastable photo-intermediate that initiates the generation of a visual electrical signal [1,12,15,24]. The decay of metarhodopsin II subsequently results in the hydrolysis of the Schiff base linkage, dissociation of the all-*trans* retinal chromophore from the opsin apoprotein, and eventual regeneration of the visual pigment through the visual (retinoid) cycle [2,7,11,26].

### 2.2. Porphyropsin (Carp Fish Rhodopsin)

**Figure 5:**
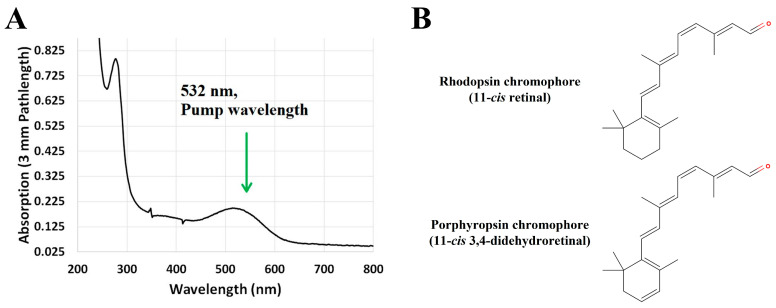
(**A**) Steady-state absorption spectrum of porphyropsin solubilized in a detergent-buffer medium. The excitation wavelength employed for pumping is indicated by the arrow. Figure is reproduced from [62]. (**B**) Chemical structures of the retinal chromophores in rhodopsin and porphyropsin, namely 11-*cis* retinal and 11-*cis* 3,4-didehydroretinal, respectively. Note the additional double bond in the terminal ionone ring of the 11-*cis* 3,4-didehydroretinal chromophore.

Similar time-resolved absorption experiments were performed on the closely related porphyropsin visual pigment, in which the 11-*cis* retinal chromophore (a derivative of vitamin A_1_) is substituted with an 11-*cis* 3,4-didehydroretinal chromophore (a derivative of vitamin A_2_) that features an extra double bond in its terminal ionone ring [10,19,20,21]. Porphyropsin is commonly found in aquatic vertebrates as an adaptation to turbid, red-shifted underwater environments, owing to its longer wavelength of peak absorption relative to rhodopsin [19,20,28]. The steady-state absorption spectrum of the porphyropsin visual pigment solubilized in a detergent-buffer medium, along with the corresponding chemical structures of the retinal chromophores in rhodopsin and porphyropsin, is displayed in Figure 5.

**Figure 6:**
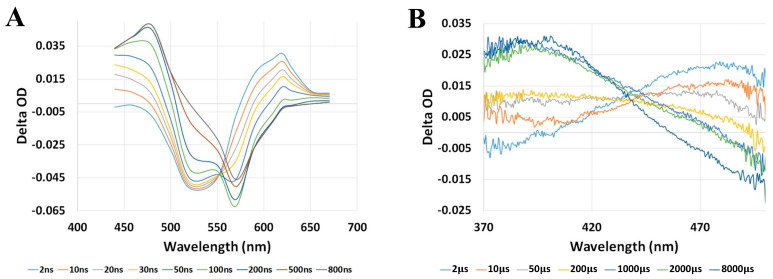
(**A**) Nanosecond transient absorption spectra of porphyropsin following optical excitation with a nanosecond laser pulse at 532 nm, illustrating the conversion of Batho (maximum absorption at ~620 nm) into Lumi (maximum absorption at ~460 nm) and subsequent formation of Meta−I (maximum absorption at ~475 nm). (**B**) Microsecond to millisecond transient absorption spectra of porphyropsin following optical excitation with a nanosecond laser pulse at 532 nm, showing the decay of Meta−I (maximum absorption at ~475 nm) and formation of Meta−II (maximum absorption at ~400 nm). Figures are reproduced with some modifications from [62].

**Figure 7:**
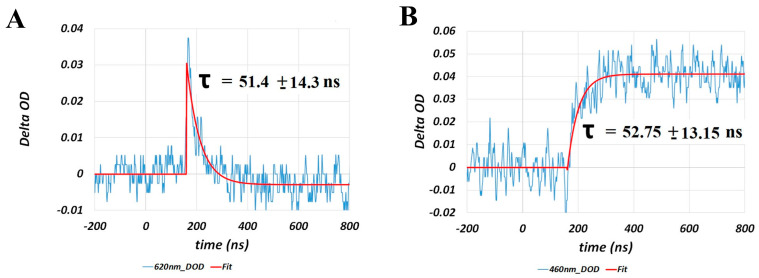
Kinetic traces and exponential fits (assuming first−order kinetics) at (**A**) 620 nm and (**B**) 460 nm, corresponding to the decay of the Batho−intermediate and formation of the Lumi−intermediate, respectively, for porphyropsin following optical excitation with a nanosecond laser pulse at 532 nm. Note that the initial high−amplitude, rapidly decaying component of the kinetic traces is due to the instrument response function of the photodetector. Figures are reproduced from [62].

The red-shifted absorption band of porphyropsin (relative to rhodopsin) shown in Figure 5A, with maximum absorption observed at ~520 nm, is due to the increase in π-conjugation, and hence electron delocalization, caused by the additional conjugated double bond in the terminal ionone ring [20], as shown in Figure 5B. Consequently, we observed significant spectral and kinetic differences in the photobleaching sequence of porphyropsin compared to that of rhodopsin following optical excitation with a nanosecond laser pulse at 532 nm, as summarized in Figure 6 and Figure 7.

The spectral and kinetic data presented in Figure 6 and Figure 7 demonstrate that the porphyropsin visual pigment undergoes a photochemical transformation analogous to that of bovine rhodopsin with respect to the sequential formation of the equivalent Batho, Lumi, and Meta-I/II photo-intermediates, which progressively display blue-shifted absorption band maxima in accordance with the conformational changes in the opsin apoprotein. Notably, we observed that the decay and formation kinetics of the equivalent Batho and Lumi photo-intermediates, respectively, were significantly faster, namely by a factor of ~3, than those recorded for bovine rhodopsin (see Figure 3) solubilized in an identical detergent-buffer medium. Similar kinetic differences were deduced for the decay and formation of the equivalent Meta-I and Meta-II photo-intermediates, respectively, based on the spectral data shown in Figure 6B, which again suggested that the photobleaching sequence proceeds at a faster rate for porphyropsin compared to bovine rhodopsin. In addition to these notable kinetic differences, we observed that the absorption band maxima of the major photo-intermediate species for porphyropsin were generally red-shifted compared to the corresponding bovine rhodopsin photo-intermediaries, which is consistent with the ~20 nm bathochromic shift observed for the steady-state absorption spectrum displayed in Figure 5A. To our knowledge, this was the first time that such spectral and kinetic differences were observed in the photobleaching sequences of the rhodopsin and porphyropsin visual pigments, which we attributed to the inherent structural differences in their constituent retinal forms, as displayed in Figure 5B.

### 2.3. Human Green-Cone Opsin

**Figure 8:**
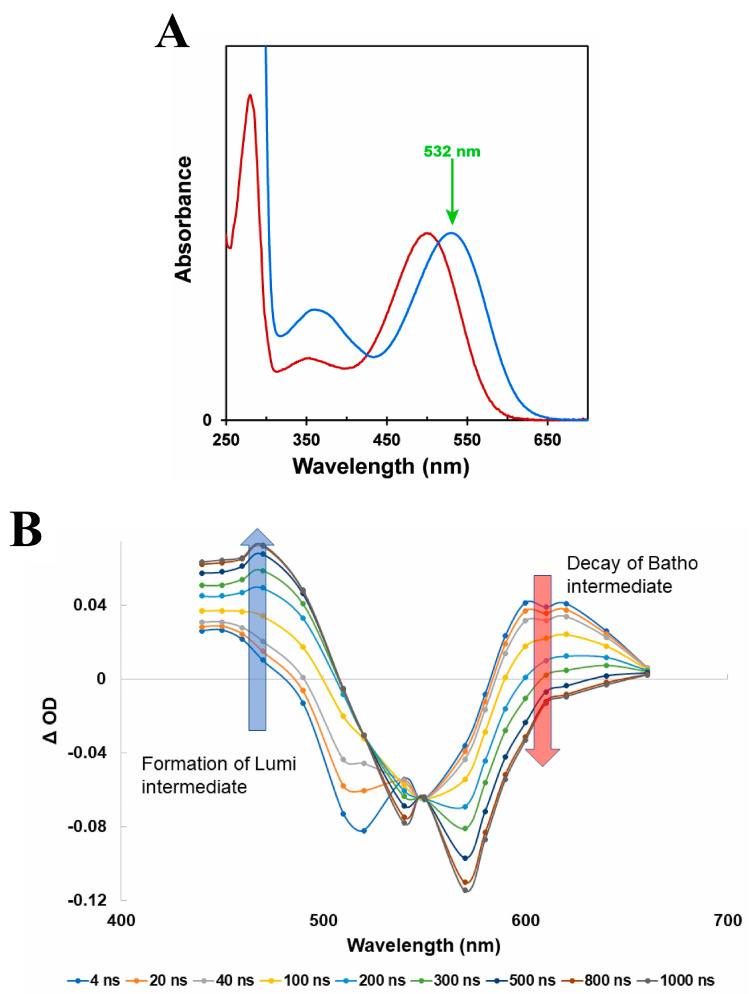

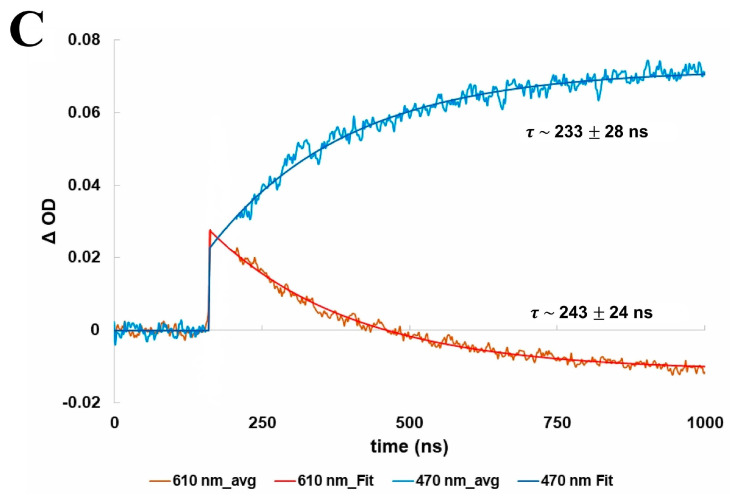
(**A**) Normalized steady−state absorption spectra of bovine rhodopsin (red) and the human green−cone visual pigment (blue) with each pigment solubilized in a detergent−buffer medium. The excitation wavelength employed for pumping is indicated by the arrow. (**B**) Nanosecond transient absorption spectra of the human green−cone visual pigment at various pump−probe delay times following optical excitation with a nanosecond laser pulse at 532 nm. (**C**) Kinetic traces and exponential fits (assuming first−order kinetics) at 610 nm (red) and 470 nm (blue), corresponding to the decay of the Batho−intermediate and formation of the Lumi−intermediate, respectively, for the human green−cone visual pigment following optical excitation with a nanosecond laser pulse at 532 nm. Note that the first ~40 ns of the recorded kinetic traces, following excitation, are excluded from the exponential fittings due to the photodetector response. Figures are reproduced with some modifications from [63].

Advances in recombinant techniques [64] have made feasible the purification and stabilization of the human green-cone visual pigment to a level suitable for use in our time-resolved absorption experiments. To that end, we recorded the nanosecond to millisecond transient absorption spectra and kinetics of the photo-intermediate states of the human green-cone visual pigment for the first time. Figure 8 displays the steady-state absorption spectrum of the human green-cone visual pigment solubilized in a detergent-buffer medium, along with the corresponding nanosecond transient absorption spectra and kinetics following optical excitation with a nanosecond laser pulse at 532 nm.

The nanosecond transient absorption spectra in Figure 8B clearly show that an equivalent Batho-intermediate, with an absorption band red-shifted relative to the human green-cone ground-state absorption band (see Figure 8A), is formed upon optical excitation of the human green-cone visual pigment. Consequently, the Batho absorption band centered at ~610 nm decays in time and results in the formation of an equivalent Lumi-intermediate with a blue-shifted absorption band maximum at ~470 nm. In addition, the kinetic data displayed in Figure 8C demonstrate, rather emphatically, that the decay and formation kinetics for the human green-cone Batho-intermediate and Lumi-intermediate, respectively, are slower by a factor of ~1.5 compared to those recorded for bovine rhodopsin (see Figure 3).

**Figure 9:**
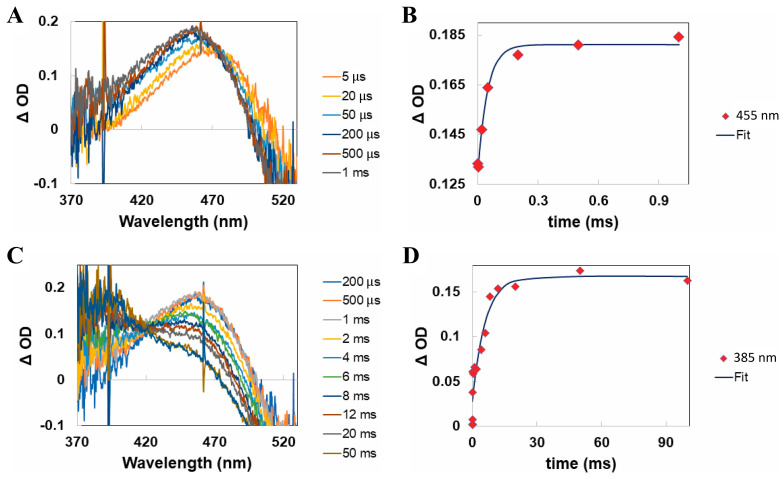
Microsecond to millisecond transient absorption spectra and kinetics of the human green−cone visual pigment following optical excitation with a nanosecond laser pulse at 532 nm. (**A**) Microsecond transient absorption spectra of the human green−cone visual pigment, illustrating the conversion of Lumi (maximum absorption at ~470 nm) into Meta−I (maximum absorption at ~455 nm). (**B**) Formation kinetics for Meta−I based on the ∆OD at 455 nm. (**C**) Millisecond transient absorption spectra of the human green−cone visual pigment, illustrating the conversion of Meta−I (maximum absorption at ~455 nm) into Meta−II (maximum absorption at ~385 nm). (**D**) Formation kinetics for Meta−II based on the ∆OD at 385 nm. Figures are reproduced with some modifications from [63].

The microsecond to millisecond transient absorption spectral data (see Figure 9A,C) clearly show the formation and decay of the equivalent Meta-I and Meta-II photo-intermediates on the microsecond and millisecond timescales, respectively, with progressively blue-shifted absorption band maxima. Similar analysis of the kinetic data displayed in Figure 9B,D further suggested that the timescales for the formation of the equivalent Meta-I and Meta-II photo-intermediates are again slower in the case of the human green-cone visual pigment by factors of ~5 and 4, respectively, compared to those for bovine rhodopsin. The extent of the Meta-I and Meta-II interconversion for the human green-cone visual pigment was also determined to be qualitatively different compared to bovine rhodopsin based on the incomplete decay of the ~455 nm absorption band, as shown in Figure 9C, which suggests only partial conversion of Meta-I to Meta-II for the human green-cone visual pigment (see Figure 4B for reference, which illustrates essentially complete metarhodopsin I/II interconversion for bovine rhodopsin). This has been previously observed during studies of the chicken red-cone visual pigment [60], and further discussion is provided in our corresponding original publication [63]. To our knowledge, this represented the first time-resolved study on the photochemical transformation of the human green-cone visual pigment and unequivocally demonstrated that its photobleaching kinetics are slower than those of bovine rhodopsin. We attributed this result to the differing primary structures of the rod and green-cone opsin apoproteins.

## 3. Discussion

**Figure 10:**
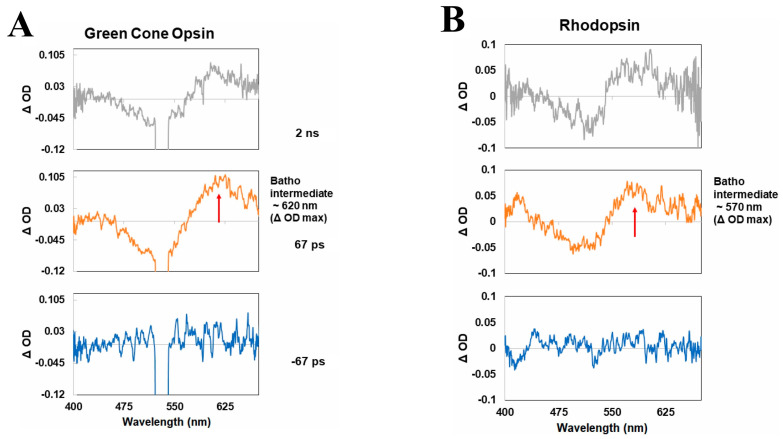
Picosecond transient absorption spectra of (**A**) the human green−cone visual pigment and (**B**) bovine rhodopsin at various pump−probe delay times following optical excitation with a picosecond laser pulse at 532 nm, illustrating the initial ultrafast formation of the first metastable Batho−intermediate within the pulse duration, along with the subsequent formation of an additional “BL intermediate” [60] for the green−cone visual pigment based on the ~10 nm blue−shift in the absorption band maximum of the Batho−intermediate. Figures are reproduced with some modifications from [63].

In this paper, we have reviewed our recent time-resolved spectroscopic studies of the rod and cone visual pigments, namely with respect to the transient molecular spectra and kinetics of their respective photobleaching sequences. We note that while we have primarily discussed the nanosecond to millisecond transient absorption spectra and kinetics of the visual pigments in this paper, picosecond spectroscopic data recorded by us [63] and others [60] suggest that an additional “BL intermediate” forms after the initial Batho-intermediate and prior to the Lumi-intermediate for specifically the cone visual pigments. This conclusion is based on a slight, ~10 nm, blue-shift in the absorption band maximum of the initial Batho-intermediate when comparing its picosecond and nanosecond transient absorption spectra (see Figure 10).

**Figure 11:**
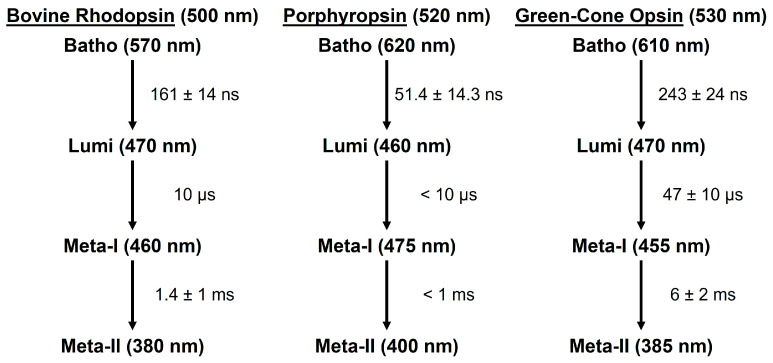
Photobleaching sequences of the bovine rhodopsin, porphyropsin, and human green-cone visual pigments at room temperature. The approximate absorption band maxima of the ground-state and photo-intermediate states are indicated in parentheses, and the corresponding decay time constants (along with the experimental error, if it was recorded) are listed for each photo-intermediate state starting with the first metastable Batho-intermediate. The noted time constants and absorption band maxima are based on our data [62,63] and supported by related research [1,15,22,23,65,66].

To facilitate comparison with the photobleaching sequences of the rhodopsin and porphyropsin visual pigments, we simply refer to the first metastable green-cone photo-intermediate (which decays in hundreds of nanoseconds) as the equivalent Batho-intermediate in our subsequent discussion. The photobleaching sequences, starting with the first metastable Batho-intermediate, of the rod and cone visual pigments described in this paper are summarized in Figure 11.

The experimental results summarized in Figure 11 clearly show that for the three visual pigments described in this paper, the porphyropsin visual pigment displays the fastest photo-intermediate formation and decay kinetics, while human green-cone opsin has the slowest photo-intermediate kinetics, at room temperature. Overall, the spectral and kinetic data (see Figure 11) suggest that the photochemical transformations of the studied rod and cone visual pigments are rather similar and generally consist of the (1) ultrafast formation (within ~1 ps at room temperature [47,48,49,50,57]) of the first metastable Batho-intermediate with a red-shifted absorption band maximum, which is then followed by (2) the sequential formation of the Lumi and Meta-I/II photo-intermediates with progressively blue-shifted absorption band maxima. Furthermore, the Batho, Lumi, and Meta-I photo-intermediates decay on approximately the nanosecond, microsecond, and millisecond timescales, respectively, for both the rod and cone visual pigments. We note that because the molecular absorption spectra for each photo-intermediate are rather broad, probing at discrete wavelengths (see Figure 3A, Figure 6A and Figure 8B) with a probe wavelength spacing of ~10–20 nm does not result in the significant loss of spectral detail, although there may be slight distortions in the precise maxima and/or shapes of the photo-intermediate absorption bands. Nevertheless, such effects do not prevent us from determining the relative locations of the photo-intermediate absorption band maxima, along with their associated formation or decay kinetics. To reiterate, cone visual pigments appear to have an additional “BL intermediate” [60,63] which directly precedes the formation of the Lumi-intermediate, but further work is required in determining whether the “BL intermediate” is conserved across the various cone visual pigments. The ordered, successive formation and decay of such photo-intermediaries is associated with increasingly large-scale conformational changes in the opsin apoprotein that facilitate G protein activation and eventual hydrolysis of the Schiff base linkage between the retinal and opsin moieties [1,15].

In addition to these general similarities, several important spectral and kinetic differences are observed in the photobleaching sequences of the rod and cone visual pigments. Comparison of the bovine rhodopsin and porphyropsin photobleaching sequences (see Figure 11) reveals that the formation and decay kinetics of the porphyropsin photo-intermediates are in general faster than those for bovine rhodopsin. Moreover, the porphyropsin ground-state and photo-intermediate absorption band maxima are generally located at longer (red-shifted) wavelengths than those for bovine rhodopsin, although the porphyropsin Lumi-intermediate specifically appears to have an absorption band maximum that is blue-shifted relative to that of lumirhodopsin. These results strongly demonstrate how substitutions in the retinal chromophore, as displayed in Figure 5B, can significantly affect the transient molecular spectra and kinetics of the resultant visual pigment, which is likely due to alterations in the retinal–opsin interaction that can accelerate or hinder conformational changes in the opsin apoprotein following photon absorption, in addition to inducing spectral shifts. Furthermore, our study of the human green-cone visual pigment, which clearly has slower photobleaching kinetics compared to bovine rhodopsin, similarly illustrates that substitutions in the opsin apoprotein (in this case, from rod opsin to green-cone photopsin [8,18]) can result in both spectral and kinetic changes. Interestingly, while the Batho-intermediate of the human green-cone visual pigment is clearly red-shifted relative to bathorhodopsin, we observed that the equivalent Lumi and Meta-I/II photo-intermediate absorption band maxima occur at comparable wavelengths as those of lumirhodopsin and metarhodopsin I/II, respectively. Hence, while both the porphyropsin and human green-cone visual pigments display similar steady-state absorption band maxima that are red-shifted relative to that of rhodopsin, the resulting effects on the photobleaching kinetics and transient photo-intermediate absorption spectra are very different.

To gain further understanding about the photochemical transformation of the cone visual pigments, we intend to conduct a similar time-resolved absorption study of the human blue-cone visual pigment, which will also be obtained through recombinant techniques [64]. The blue-cone visual pigment is structurally and spectrally (see Figure 1B for reference) the most distinct of the human cone visual pigments, sharing only ~40% similarity in its blue-cone photopsin primary structure with those of the red- and green-cone photopsins (which share ~96% similarity in contrast) [2,8,18]. To that end, we expect that detailed understanding of the blue-cone photobleaching sequence will offer critical insight about cone-mediated vision, namely the role of ground-state and/or photo-intermediate spectral shifts in determining the resultant photobleaching kinetics. For example, one possible interpretation of the photobleaching kinetic differences (see Figure 11) between rhodopsin and the human green-cone visual pigment is that the larger spectral shift between the absorption band maxima of the ground-state and Meta-II photo-intermediate forms (~120 nm for rhodopsin and ~145 nm for green-cone opsin) confers more significant conformational changes in the opsin apoprotein following photon absorption and prior to retinal dissociation, which results in the observed slower photobleaching kinetics for the human green-cone visual pigment. In the case of the human blue-cone visual pigment, the chromophore is still 11-*cis* retinal, and we therefore expect the Meta-II photo-intermediate to similarly display an absorption band maximum near 380 nm [67], which is blue-shifted from the human blue-cone ground-state absorption band maximum (located at ~425 nm [8]) by only ~45 nm. Based on the above proposition, this may result in faster photobleaching kinetics for the blue-cone visual pigment. It will also be interesting to record any differences in the transient human blue-cone photo-intermediate spectra, and/or spectral shifts, with respect to the rhodopsin and human green-cone visual pigment photobleaching sequences (see Figure 11) and determine whether these can also be correlated with significant kinetic differences. Finally, we note that the human blue-cone visual pigment appears to have the smallest steady-state absorption spectrum FWHM bandwidth (relative to rhodopsin and the other human cone visual pigments), as shown in Figure 1B, which may offer additional insight regarding its unique retinal–opsin interaction and corresponding photo-intermediate kinetics.

**Figure 12:**
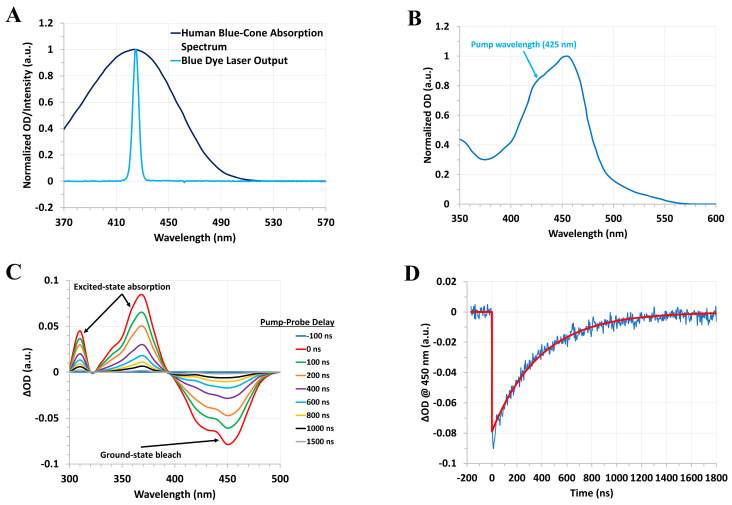
(**A**) Normalized absorption spectrum (dark blue) of the human blue−cone visual pigment (spectrum is plotted based on the data in [8]) and normalized output spectrum (light blue) of the homebuilt blue dye laser. (**B**) Normalized absorption spectrum of [Ru(bpy)_3_]Cl_2_∙6H_2_O dissolved in distilled water. The ~425 nm pump wavelength of the blue dye laser is indicated by the arrow. (**C**) Nanosecond transient absorption spectra of [Ru(bpy)_3_]Cl_2_∙6H_2_O dissolved in distilled water (steady−state OD ~1 a.u. at 425 nm with a 1 cm path length) at various pump−probe delay times following excitation at 425 nm with the blue dye laser. These spectra were derived from the first−order exponential fits of the transients recorded in the 300–500 nm wavelength range. (**D**) Kinetic trace and exponential fit (assuming first−order kinetics) at a probe wavelength of 450 nm, which corresponds to the bleach and subsequent recovery of the ground−state population with an estimated lifetime of ~400 ns. Similar time constants (within the experimental error) are obtained for the decay of the excited−state (triplet–triplet) absorption bands centered at ~310 nm and 370 nm. Note that the initial high−amplitude, rapidly decaying component of the kinetic trace is due to the instrument response function of the photodetector.

For our forthcoming studies of the human blue-cone visual pigment, we have developed a homebuilt blue dye laser system consisting of the laser dye stilbene 420 dissolved in methanol (typically at a concentration of ~0.3 mg/mL in a 1 cm quartz cuvette), which is pumped by the ~50 mJ third harmonic, ~355 nm, output of our Q-switched Nd:YAG nanosecond system. This setup enables the generation of ~10 ns pulses at ~425 nm with a typical pulse energy of ~1–2 mJ. To illustrate the utility of our homebuilt blue dye laser as an excitation source for pump–probe experiments, we recorded the transient absorption spectra and kinetics of [Ru(bpy)_3_]Cl_2_∙6H_2_O, which absorbs in a similar wavelength region as the human blue-cone visual pigment and displays a metal-to-ligand charge transfer (MLCT) excited triplet state lifetime of hundreds of nanoseconds [68], thus serving as a useful test sample for our blue dye laser pump–probe system. Figure 12 displays the steady-state absorption spectrum of the human blue-cone visual pigment and the output spectrum of our blue dye laser, along with the steady-state and transient absorption spectra and kinetics of [Ru(bpy)_3_]Cl_2_∙6H_2_O following excitation with our homebuilt blue dye laser.

We have also employed this homebuilt blue dye laser system to initiate and study the photochemistry of other biomolecules such as chlorophyll A. Based on our preliminary results (see Figure 12), which were obtained under similar experimental conditions as those employed for the rod and cone visual pigments (with respect to the steady-state OD of the sample at the pump wavelength), we expect to be able to record the nanosecond to millisecond transient molecular spectra and kinetics of the blue-cone photo-intermediates following pulsed excitation at ~425 nm, thereby enabling a detailed comparison of the rhodopsin, green-cone, and blue-cone visual pigment photobleaching sequences. Substitution of the stilbene dye gain medium with another dye, such as rhodamine 6G, will additionally allow us to generate intense orange pulses suitable for future time-resolved studies of the human red-cone visual pigment, thus providing a comprehensive overview of the photobleaching sequences for the various cone visual pigments.

## 4. Materials and Methods

### 4.1. Nanosecond Transient Absorption System

We have described our nanosecond transient absorption experimental system, along with the corresponding data analysis procedure, in detail previously [62,63]. Briefly, the second harmonic, ~532 nm, output of a Q-switched Nd:YAG nanosecond laser system (New Wave Research, Fremont, CA, USA, Model: Tempest-20) is employed for optically pumping the visual pigments. The FWHM pulse width is ~8 ns for the pump (excitation) pulses with ~100 mJ per pulse. Two separate Xenon flashlamps are employed as optical probes that are focused and spatially overlapped with the ~532 nm pump pulse at the sample. The first Xenon flashlamp probe has a long, ~100 µs, FWHM pulse duration, and its broad, ~4 µs, region of nearly constant, peak flash emission is synchronized with the arrival of the pump laser pulse at the sample. Changes in the intensity of the Xenon flashlamp (after interrogating the sample) were monitored, as a function of the pump–probe delay time, using a photomultiplier tube (Hamamatsu, Bridgewater, NJ, USA, Model: R928) situated at the focal plane of a monochromator (Jarrell Ash, Boston, MA, USA, Model: 82-410), which can be scanned to change the probe wavelength. The photomultiplier voltage was recorded in time with a fast oscilloscope (Tektronix, Beaverton, OR, USA, Model: TDS3052B) with an input impedance of 50 Ω to enable nanosecond temporal resolution, and the overall rise time of the PMT-oscilloscope detection system is a few nanoseconds (see Figure S1 for the PMT temporal response due to the ~532 nm pump pulse). Comparison of the photomultiplier voltage before and after the arrival of the pump pulse enables computation of ∆OD as a function of time for a given probe wavelength, and in turn, scanning the probe wavelength allows us to record time-resolved absorption spectra for pump–probe delays ranging from a few nanoseconds up to a few microseconds.

**Figure 13:**
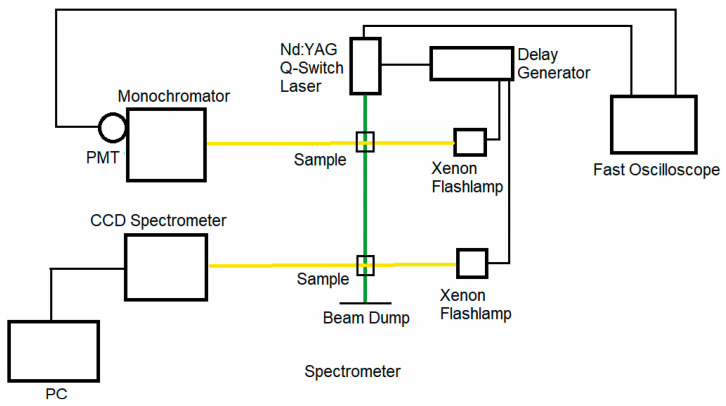
Schematic representation of the nanosecond to millisecond transient absorption experimental system, where the green and yellow lines represent the pump and probe beams, respectively. Synchronization between all subsystems is provided through the flashlamp and Q-switch trigger signals provided by the pump laser unit. Figure is reproduced from [63].

The second Xenon flashlamp probe has a significantly shorter, ~200 ns, FWHM pulse duration, and the probe pulse is electronically delayed with respect to the pump pulse by means of a digital delay generator (Princeton Instruments, Trenton, NJ, USA, Model: DG 535). A portable CCD spectrometer (B&W Tek, Plainsboro, NJ, USA) or monochromator (Acton Research Corporation, Acton, MA, USA, Model: SpectraPro-150) coupled with a water-cooled CCD camera is used to record the probe spectrum with and without the pump pulse, which enables computation of the ∆OD spectrum at a given pump–probe delay time. This probe is employed for pump–probe delays ranging from a few microseconds up to several milliseconds. A schematic representation of our nanosecond pump–probe experimental system is shown in Figure 13.

### 4.2. Picosecond Transient Absorption System

**Figure 14:**
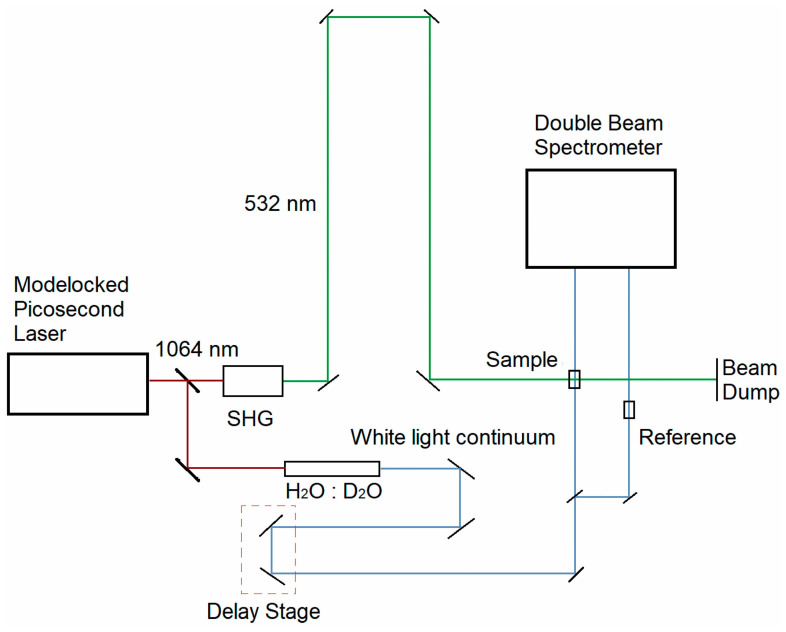
Schematic representation of the picosecond transient absorption experimental system, where the green and blue lines represent the pump and probe beams, respectively. Figure is reproduced from [63].

We have described our picosecond transient absorption experimental system, along with the corresponding data analysis procedure, in detail previously [63]. Briefly, a portion of the ~1064 nm output of a passively mode-locked Nd:YVO_4_ picosecond laser system (Ekspla, Vilnius, Lithuania, Model: PL2230) is passed through a nonlinear crystal to generate the corresponding second harmonic, ~532 nm, output used for optically pumping the visual pigments. The ~532 nm pump pulse width is ~28 ps with ~4–5 mJ per pulse. The remaining portion of the ~1064 nm fundamental beam was focused into a H_2_O/D_2_O cell to generate a visible, broadband supercontinuum that was subsequently divided into probe and reference beams. The broadband probe beam was focused and spatially overlapped with the ~532 nm pump beam at the sample. The probe and reference beams were then directed into a monochromator (Acton Research Corporation, Model: SpectraPro-150) coupled with a thermoelectrically cooled 2-D CCD camera (Princeton Instruments, Model: PIXIS:400) that recorded the probe and reference spectra over the wavelength range of ~225–750 nm. The probe and reference spectra were recorded with and without the ~532 nm pump pulse, which enables computation of the ∆OD spectrum at a given pump–probe delay time. A linear translation delay stage was employed for temporally delaying the probe pulse with respect to the pump pulse for pump–probe delay times ranging from roughly −100 ps up to +4 ns. A schematic representation of our picosecond pump–probe experimental system is shown in Figure 14.

### 4.3. Rod and Cone Visual Pigments

The complete details regarding the preparation, purification, and solubilization of the rod and cone visual pigments described in this paper are provided in our original publications [62,63]. Briefly, the bovine rhodopsin and porphyropsin (carp fish rhodopsin) visual pigments were extracted from rod outer segments (ROS) and solubilized in a detergent-buffer medium to ensure stability for experiments. The human green-cone visual pigment was prepared as described in [64] and similarly solubilized in a detergent-buffer medium. All visual pigments were kept frozen in the dark prior to their usage in experiments. The steady-state absorption spectra of the visual pigment samples were recorded with a Cary 50 Bio UV–Vis spectrophotometer or Shimadzu 1601 UV–Vis spectrophotometer. For the time-resolved experiments, visual pigment samples were prepared to have a steady-state OD of ~0.5–1 a.u. (10 mm path length) at their ground-state absorption band maximum, and ~10–15 µL of the sample was placed in a microchannel quartz cuvette with 1 mm and 10 mm pump and probe path lengths, respectively. All steady-state and time-resolved measurements were performed at room temperature under dim red light, and the sample was replaced after every pump laser shot to minimize photobleaching effects.

## 5. Conclusions

This review paper has presented an overview of our previous and recent ultrafast spectroscopic studies of rod and cone visual pigments. We have described, in some detail, the photochemical transformation of the bovine rhodopsin, porphyropsin, and human green-cone visual pigments following optical excitation with picosecond and nanosecond laser pulses, along with the significant spectral and kinetic differences observed between their respective photobleaching sequences. These results demonstrate the influence of the visual pigment molecular structure on the resultant photobleaching sequence, namely how substitutions in the retinal chromophore or opsin apoprotein can greatly influence and alter the transient molecular spectra and/or kinetics of the visual pigment photo-intermediates. To the best of our knowledge, this review paper provides the first direct comparison of the transient photo-intermediate absorption spectra and kinetics for these three visual pigments on the picosecond to millisecond timescales, which should motivate additional time-resolved or structure–function studies of these and related visual pigments, particularly other cone visual pigments. We expect that our forthcoming time-resolved studies of the human blue-cone and red-cone visual pigments will enable us to further elucidate the role of ground-state and photo-intermediate spectral shifts in determining the corresponding photobleaching kinetics of the rod and cone visual pigments, in addition to providing greater insight regarding the molecular basis for cone-mediated color vision. These results will altogether enable elucidation of important functional differences between the rod and cone phototransduction mechanisms.

## Data Availability

All data are either cited and/or included in the main text.

## Supplementary Materials

The following supporting information can be downloaded at: https://www.mdpi.com/article/10.3390/molecules28155829/s1, Figure S1: Temporal response of the PMT due to the ~532 nm pump (excitation) pulse.
