# Supplementary material for: Ultrafast Transient Absorption Spectra and Kinetics of Rod and Cone Visual Pigments
Source: Molecules. 2023 Aug 2;28(15):5829. doi: 10.3390/molecules28155829 (PMC10421382; doi:10.3390/molecules28155829)
Supplement: Supplementary file 1 [file molecules-28-05829-s001.zip › molecules-2517915-supplementary.pdf]

SUPPLEMENTARY INFORMATION

Ultrafast Transient Absorption Spectra and Kinetics of Rod and Cone Visual Pigments

Arjun Krishnamoorthi [1], Keyvan Khosh Abady [1], Dinesh Dhankhar [1,2] and Peter M. Rentzepis [1,*]

[1] Department of Electrical and Computer Engineering, Texas A&M University, College Station, TX 77843, USA

[2] Present address: Thermo Fisher Scientific, Hillsboro, OR 97124, USA

* Correspondence: prentzepis@tamu.edu

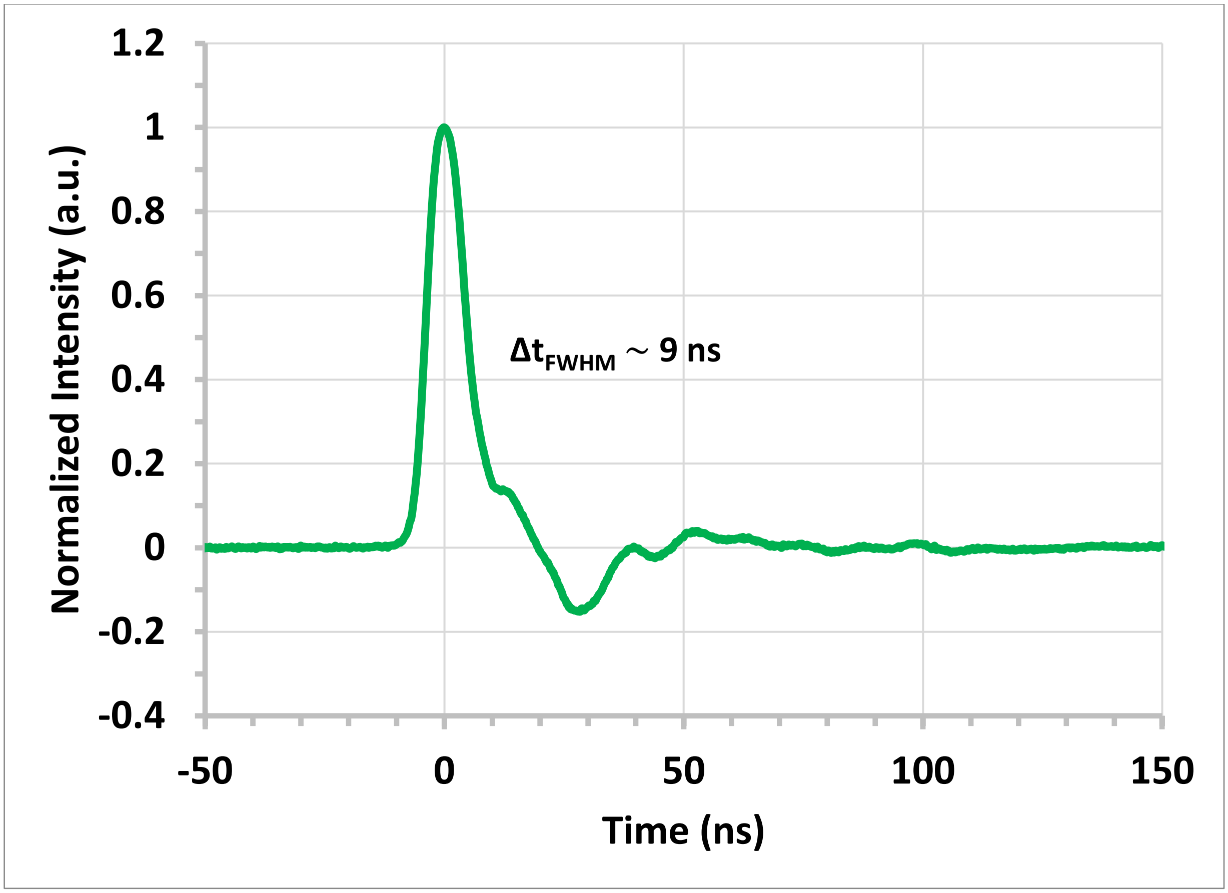

Figure S1. Temporal response of the PMT due to the ~ 532 nm pump (excitation) pulse.
